# Case Report: Fibromatosis colli in a neonate

**DOI:** 10.4103/0971-3026.59753

**Published:** 2010-02

**Authors:** S Smiti, Naveen M Kulkarni, Jyoti Singh

**Affiliations:** Department of Radio-diagnosis and Imaging, Kasturba Medical College, Manipal, Karnataka, India

**Keywords:** Fibromatosis colli, sternocleidomastoid, infant

## Abstract

Fibromatosis colli or pseudotumor of infancy of the sternocleidomastoid muscle is a rare cause of a benign neck mass in neonates and infants. If diagnosed correctly, it can be managed conservatively, and unnecessary investigations can be avoided.

## Introduction

Fibromatosis colli is a condition in which there is diffuse enlargement of the sternocleidomastoid muscle, usually in infancy.[1] Though the exact etiology is not known, it is most likely due to birth trauma.[2] It is one of the causes of congenital torticollis. Though USG is the imaging modality of choice, cross sectional imaging with CT scan or MRI may sometimes be required to further characterize the disease and to know the extent of involvement. Real time USG shows synchronous motion of the mass with the sternocleidomastoid muscle, thus confirming the diagnosis. We present a case report where fibromatosis colli was diagnosed using USG, in an infant.

## Case Report

A 3 1/2-week-old neonate was referred to the radiology department for USG of a neck swelling on the left side that had been noticed by the parents 2 weeks ago. The swelling was firm to hard in consistency and was not warm to touch. The patient was afebrile. There was restriction of neck movements on the affected side. The parents reported that the child had had a forceps delivery.

**Figure 1 (a,b):**
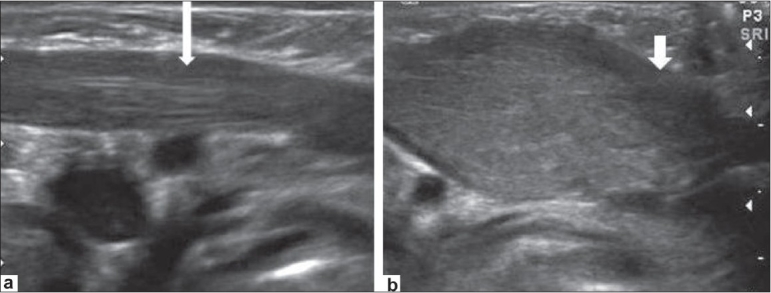
Longitudinal USG images of the neck show fusiform enlargement of the left sternocleidomastoid muscle (short arrow in b), with normal muscle on the right (long arrow in a)

**Figure 2:**
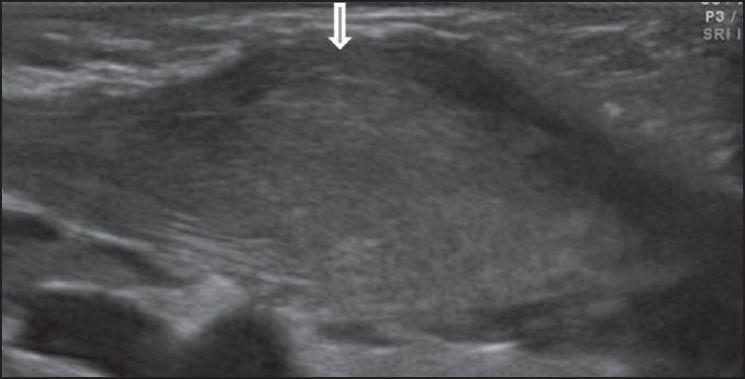
Longitudinal USG of the neck shows an enlarged sternocleidomastoid muscle with heterogenous echotexture (arrow)

USG showed a thickened sternocleidomastoid muscle on the left; it had a fusiform appearance and heterogenous echotexture. The fibrillar structure of the muscle fibers was however maintained [Figure 1]. In comparison, the right sternocleidomastoid muscle appeared normal [Figure 2]. There was no cervical lymphadenopathy. Based on these USG features and the clinical findings, a diagnosis of fibromatosis colli or pseudotumor of the sternocleidomastoid muscle was considered.

Physiotherapy was started and the swelling showed a slight decrease in size after 3 weeks, with the neck movements returning to near normal.

## Discussion

It typically presents with a neck swelling at 2-4 weeks of birth, most commonly following a difficult delivery (vacuum extraction or forceps delivery). The diagnosis can be made on USG, which shows spindle-shaped thickening of the sternocleidomastoid muscle on the affected side in contrast to the normal contralateral side. There is no cervical lymphadenopathy and no vascular invasion or bony involvement as may be seen with other neck masses. Torticollis can develop in around 20% of cases.[3] Bilateral sternocleidomastoid tumors of infancy, though extremely rare, have also been described.[4] Treatment is symptomatic, with physiotherapy and neck stretching exercises. The swelling regresses over a period of time, with complete disappearance by 4-6 months. The differential diagnosis of solid tumors in this situation includes rhabdomyosarcoma and neuroblastoma in which, in addition to a neck mass, there can be enlarged cervical lymph nodes, vascular encasement, or invasion of surrounding structures.

USG can correctly identify this entity in almost all the cases though CT scan and MRI features have also been described.[1] On CT scan, the sternocleidomastoid muscle appears diffusely enlarged, isodense in attenuation.[1] MRI features include decreased signal intensity of the mass on T2W images as compared to gradient-recalled T1W images, because of the presence of fibrous tissue.[5] The extent of involved muscle is better delineated with MRI than with USG. The cytologic features include bland-appearing fibroblasts and atrophic skeletal muscle, along with muscle giant cells and bare nuclei.[6]

To conclude, fibromatosis colli is a relatively rare cause of neck swelling in neonates and infants and the radiologist must be aware of its imaging features in order to differentiate it from other neck masses.

## References

[1] Crawford SC, Harnsberger HR, Johnson L (1988). J Richard Aoki and Jim Alley - Fibromatosis colli of infancy: CT and sonographic findings. AJR Am J Roentgenol.

[2] McQueen WJ, Johnsons JT, Edwards PA (1980). Fibromatosis colli -a case report. Otolaryngol Head Neck Surg.

[3] Schneble F Fibromatosis colli - sternocleidomastoid pseudotumor of infancy. PedRad [serial online].

[4] Kumar V, Prabhu BV, Chattopadhyaya A, Nagendhar MY (2003). Bilateral sternocleidomastoid tumour of infancy. Int J Pediatric Otorhinolaryngol.

[5] Ablin DS, Jain K, Howell L, Steel D (1998). West-Ultrasound and MR imaging of Fibromatosis Colli Pediatr Radiol.

[6] Sharma S, Mishra K, Khanna G (2003). Fibromatosis colli in infants- a cytologic study of eight cases. Acta Cytol.

